# Leukotriene D_4_ and Interleukin-13 Cooperate to Increase the Release of Eotaxin-3 by Airway Epithelial Cells

**DOI:** 10.1371/journal.pone.0043544

**Published:** 2012-08-31

**Authors:** Véronique Provost, Anick Langlois, François Chouinard, Marek Rola-Pleszczynski, Jamila Chakir, Nicolas Flamand, Michel Laviolette

**Affiliations:** 1 Centre de recherche de l'Institut universitaire de cardiologie et de pneumologie de Québec, Université Laval, Québec, QC, Canada; 2 Unité d'immunologie, Faculté de médecine, Université de Sherbrooke, Sherbrooke, QC, Canada; Université Libre de Bruxelles, Belgium

## Abstract

**Introduction:**

Airway epithelial cells play a central role in the physiopathology of asthma. They release eotaxins when treated with T_H_2 cytokines such as interleukin (IL)-4 or IL-13, and these chemokines attract eosinophils and potentiate the biosynthesis of cysteinyl leukotrienes (cysLTs), which in turn induce bronchoconstriction and mucus secretion. These effects of cysLTs mainly mediated by CysLT_1_ and CysLT_2_ receptors on epithelial cell functions remain largely undefined. Because the release of inflammatory cytokines, eotaxins, and cysLTs occur relatively at the same time and location in the lung tissue, we hypothesized that they regulate inflammation cooperatively rather than redundantly. We therefore investigated whether cysLTs and the T_H_2 cytokines would act in concert to augment the release of eotaxins by airway epithelial cells.

**Methods:**

A549 cells or human primary bronchial epithelial cells were incubated with or without IL-4, IL-13, and/or LTD_4_. The release of eotaxin-3 and the expression of cysLT receptors were assessed by ELISA, RT-PCR, and flow cytometry, respectively.

**Results:**

IL-4 and IL-13 induced the release of eotaxin-3 by airway epithelial cells. LTD_4_ weakly induced the release of eotaxin-3 but clearly potentiated the IL-13-induced eotaxin-3 release. LTD_4_ had no effect on IL-4-stimulated cells. Epithelial cells expressed CysLT_1_ but not CysLT_2_. CysLT_1_ expression was increased by IL-13 but not by IL-4 and/or LTD_4_. Importantly, the upregulation of CysLT_1_ by IL-13 preceded eotaxin-3 release.

**Conclusions:**

These results demonstrate a stepwise cooperation between IL-13 and LTD_4_. IL-13 upregulates CysLT_1_ expression and consequently the response to cysLTs This results in an increased release of eotaxin-3 by epithelial cells which at its turn increases the recruitment of leukocytes and their biosynthesis of cysLTs. This positive amplification loop involving epithelial cells and leukocytes could be implicated in the recruitment of eosinophils observed in asthmatics.

## Introduction

Asthma is characterized by airway inflammation and remodeling processes, leading to bronchial hyperresponsiveness [1]. Airway epithelial cells likely play a central role in the pathophysiology of asthma given their ability to release numerous soluble mediators implicated in the inflammatory response [1]. The T_H_2 cytokines interleukin (IL)-4 and IL-13 are found in the bronchial fluids of asthmatic subjects and stimulate airway epithelial cells to release significant levels of eotaxins, which are potent chemotactic factors for eosinophils [2], [3].

Eotaxins represent a group of chemokines consisting of eotaxin-1 (CCL11), eotaxin-2 (CCL24) and eotaxin-3 (CCL26) [4]. The production of the different eotaxins is cell-type specific. Eotaxin-1 is secreted by eosinophils, macrophages, lymphocytes, fibroblasts, smooth muscle and endothelial cells, whereas eotaxin-2 and eotaxin-3 are mainly released by epithelial and endothelial cells [4]. Among these cell type, epithelial cells are the major source of eotaxins and principally release high levels of eotaxin-3 [2], [5], [6]. Moreover, the release of eotaxins is differentially modulated by cytokines. The T_H_2 cytokines IL-4 and IL-13 enhance the secretion of all eotaxins, whereas the T_H_1 cytokines interferon-γ and tumor necrosis factor-α exclusively promote the release of eotaxin-1 [7], [8].

Blood eosinophils migrate in the tissue under the action of potent and specific chemoattractants such as 5-oxo-6,8,11,14-eicosatetraenoic acid and eotaxin-1 [3], [9]. Once in the mucosa, eosinophils generate and release soluble mediators that activate resident cells. Notably, eosinophils are an important source of cysteinyl leukotrienes (cysLTs), which induce bronchoconstriction and mucus secretion, and promote eosinophil trafficking into the bronchial mucosa [10]. The effects of cysLTs on epithelial functions are mostly uncharacterized.

CysLTs mediate most of their biological effects through at least two distinct receptors, namely CysLT_1_ and CysLT_2_ [11], [12]. CysLT_1_ is expressed in several tissues, on myeloid and smooth muscle cells, and T_H_2 cytokines enhance its expression [13]–[16]. A recent study also showed that airway epithelial cells expressed CysLT_1_ and this expression was increased in asthmatic individuals [17]. CysLT_2_ is expressed on eosinophils, macrophages, endothelial and smooth muscle cells, in the heart, brain and the adrenals [11].

Given that airway epithelial cells are an important source of eotaxins and are activated by Th2 cytokines and cysLTs, we hypothesized that the incubation of epithelial cells with both cysLTs and Th2 cytokines would enhance the release of eotaxins. The results presented in this study demonstrate a cooperation between LTD_4_ and IL-13 for the release of eotaxin-3 by airway epithelial cells.

## Results

### IL-13 stimulates airway epithelial cells to release eotaxin-2 and eotaxin-3

**Figure 1:**
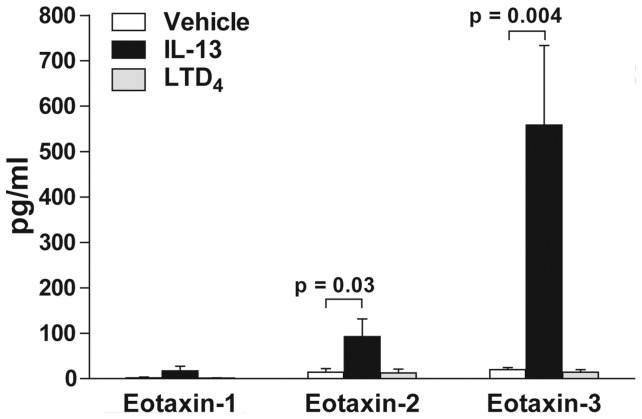
Effect of IL-13 on the release of eotaxins from airway epithelial cells. Airway epithelial cells were treated with vehicle (open bars), 10 ng/ml IL-13 (closed bars), or 100 nM LTD_4_ (gray bars) for 24 hours. Incubations were stopped by removing the supernatants and eotaxin-1, eotaxin-2, and eotaxin-3 were quantified as described in *Material and Methods*. The data represent the mean (± SEM) of four independent experiments.

In a first series of experiments, we evaluated the effect of IL-13 and LTD_4_ on the release of eotaxins following a 24 hours incubation of A549 airway epithelial cells with these mediators. Vehicle- or LTD_4_-treated airway epithelial cells released minimal amounts of eotaxin-1, eotaxin-2, and eotaxin-3. In contrast, the treatment of airway epithelial cells with IL-13 induced a small accumulation of eotaxin-2 and a more substantial accumulation of eotaxin-3, without stimulating that of eotaxin-1 (Figure 1).

**Figure 2:**
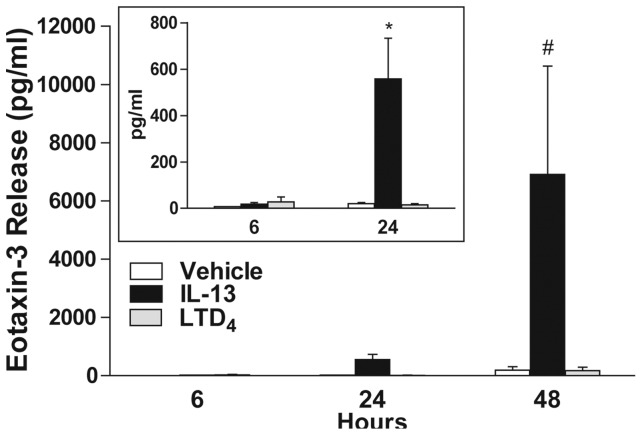
Kinetic of eotaxin-3 release from airway epithelial cells. Airway epithelial cells were incubated with vehicle (open bars), 10 ng/ml IL-13 (closed bars), or 100 nM LTD_4_ (gray bars) for different times. Incubations were stopped by removing the supernatants and eotaxin-3 was quantified as described in *Material and Methods*. The data represent the mean (± SEM) of four independent experiments. Insert represents a zoom of the 6 and 24 hours data points. * p = 0.002 vs 6 hours, # p = 0.003 vs 24 hours.

Since IL-13 greatly induced eotaxin-3 release (Figure 1) and that human epithelial cells mainly produce eotaxin-3 [2], [5], [6], we performed another series of experiments to further characterize the accumulation of eotaxin-3 in the supernatant of A549 cells treated with IL-13. The IL-13-induced eotaxin-3 accumulation was time-dependent. Eotaxin-3 was barely detectable after 6 hours (Figure 2 insert) but was observed after 24 hours and 48 hours of stimulation (Figure 2). Noteworthy, the accumulation of eotaxin-3 in the incubation media of IL-13-treated cells at 48 hours was greater than that observed at 24 hours by one order of magnitude. In these experiments and in agreement with the data presented in Figure 1, LTD_4_ alone did not induce the production or at least the accumulation of eotaxin-3 by airway epithelial cells. We did not perform experiments at longer time points, given the cells were forming multilayers. All together, these results show that IL-13 stimulated the production of eotaxin-3 from airway epithelial cells in a time-dependent manner.

### LTD_4_ potentiates the IL-13-mediated release of eotaxin-3

**Figure 3:**
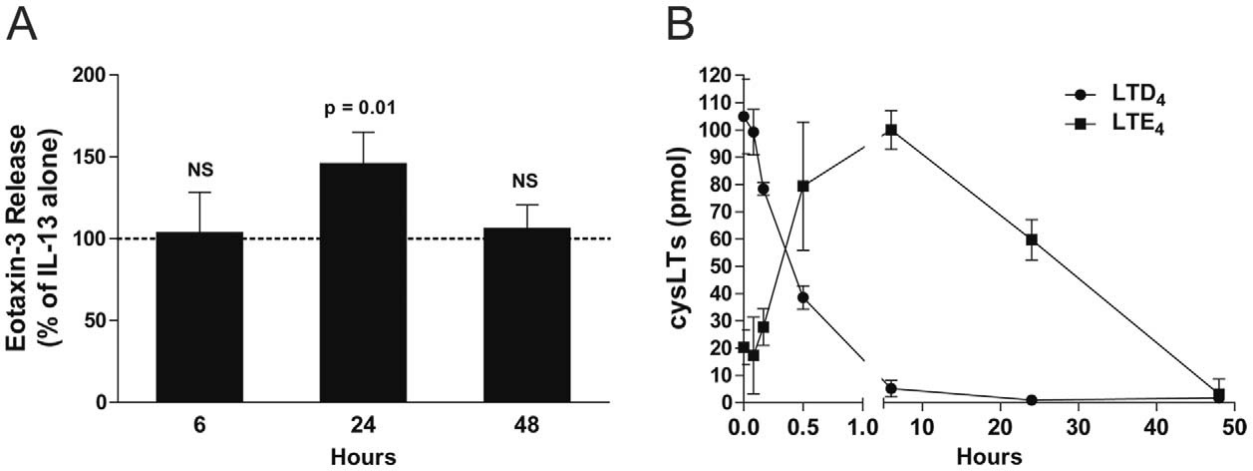
Effect of LTD_4_ on the IL-13-induced release of eotaxin-3 by airway epithelial cells. **A**) Airway epithelial cells were incubated with 10 ng/ml IL-13 alone or in combination with 100 nM LTD_4_ for up to 48 hours. Incubations were stopped by removing the supernatants and eotaxin-3 was quantified as described in *Material and Methods*. Data are expressed as % of IL-13-treated cells for each incubation period. The data represent the mean (± SEM) of four independent experiments. NS = not significant. **B**) Airway epithelial cells were incubated with 50 ng LTD_4_ for up to 48 hours. Incubations were stopped by adding one volume of cold incubation buffer. Samples were harvested then processed for the analysis of LTD_4_ and LTE_4_ by reverse-phase HPLC as described in *Material and Methods*. Data are the mean (± SEM) of three independent experiments, each performed in duplicate.

When compared to pro-inflammatory cytokines or toll-like receptor agonists, LTs are weak inducers of cytokine and chemokine production and release [18]. However, they amplify the effects of cytokines and toll-like receptors agonists on gene expression in numerous experimental conditions [19]–[28]. We consequently investigated whether LTD_4_ could potentiate the effect of IL-13 on eotaxin-3 release by airway epithelial cells. As shown in Figure 3, the incubation of airway epithelial cells with the combination of IL-13 and LTD_4_ amplified the release of eotaxin-3 induced by IL-13 by 45%. This amplification could however only be observed at 24 hours when eotaxin-3 accumulation remain moderate. The lack of effect of LTD_4_ at 48 hours prompted us to perform additional experiments in which we assessed the half-life of LTD_4_. As shown in Figure 3B, LTD_4_ added to A549 cells was rapidly metabolized into LTE_4_, with a half-life of ∼22 min. In contrast, LTE_4_ remained in our samples for a much longer period. However, LTE_4_ did not mimic the effect of LTD_4_ on IL-13-treated A549 cells (data not shown).

### Airway epithelial cells and primary human bronchial epithelial cells express CysLT_1_ but not CysLT_2_

**Figure 4:**
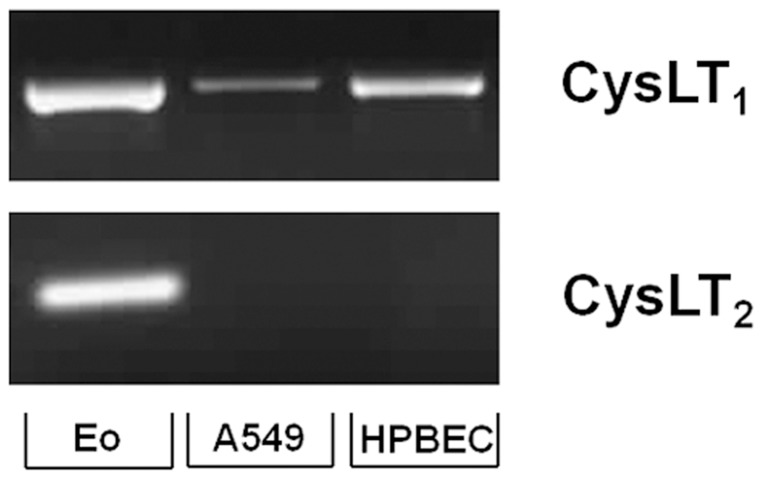
Expression of CysLT_1_ and CysLT_2_ by airway epithelial cells. Total mRNA was extracted from resting airway epithelial cells (A549), human primary bronchial epithelial cells (HPBEC) and freshly isolated human eosinophils (Eo). The expression of CysLT_1_ and CysLT_2_ mRNA was then analyzed by RT-PCR as described in *Material and Methods*. The data presented are a typical result of three independent experiments for eosinophils and airway epithelial cells, and four independent experiments for HPBEC.

Given that LTD_4_ preferentially activates CysLT_1_ and CysLT_2_ [11], [12], we next addressed if these receptors were involved in the effects of LTD_4_ on eotaxin-3 release. We first screened whether CysLT_1_ and CysLT_2_ were expressed by resting airway epithelial or primary human bronchial epithelial cells. Eosinophils were used as positive controls since they express both receptors [29], [30]. The expression pattern of CysLT receptors of airway epithelial and primary human bronchial epithelial cells were similar, as pictured in Figure 4. Indeed, only the mRNA for CysLT_1_ was detected in both cell types, indicating that the effect of LTD_4_ on eotaxin-3 release is likely the consequence of CysLT_1_ activation.

### LTD_4_ does not modulate the IL-4-induced eotaxin-3 release from airway epithelial cells

Since IL-4 is also known to induce the release of eotaxin-3 by airway epithelial cells [2] and that IL-4 and IL-13 share common receptor subunits [31], we also tested the effect of IL-4 alone or in combination with LTD_4_ on eotaxin release after a 24 hr incubation. Similarly to IL-13, IL-4 induced the release of eotaxin-1 (28.3±15.3 pg/ml p = 0.04), eotaxin-2 (124.3±50 pg/ml p = 0.05) and eotaxin-3 (1694±638.4 pg/ml p = 0.0006). As opposed to IL-13-stimulated cells (Figure 3), the addition of LTD_4_ to airway epithelial cells did not modulate the release of eotaxins induced by IL-4 (data not shown). Collectively, these results suggest that LTD_4_ enhances the release of eotaxin-3 by airway epithelial cells activated by IL-13 but not by IL-4.

### Differential effect of IL-4 and IL-13 on CysLT_1_ expression by airway epithelial cells

**Figure 5:**
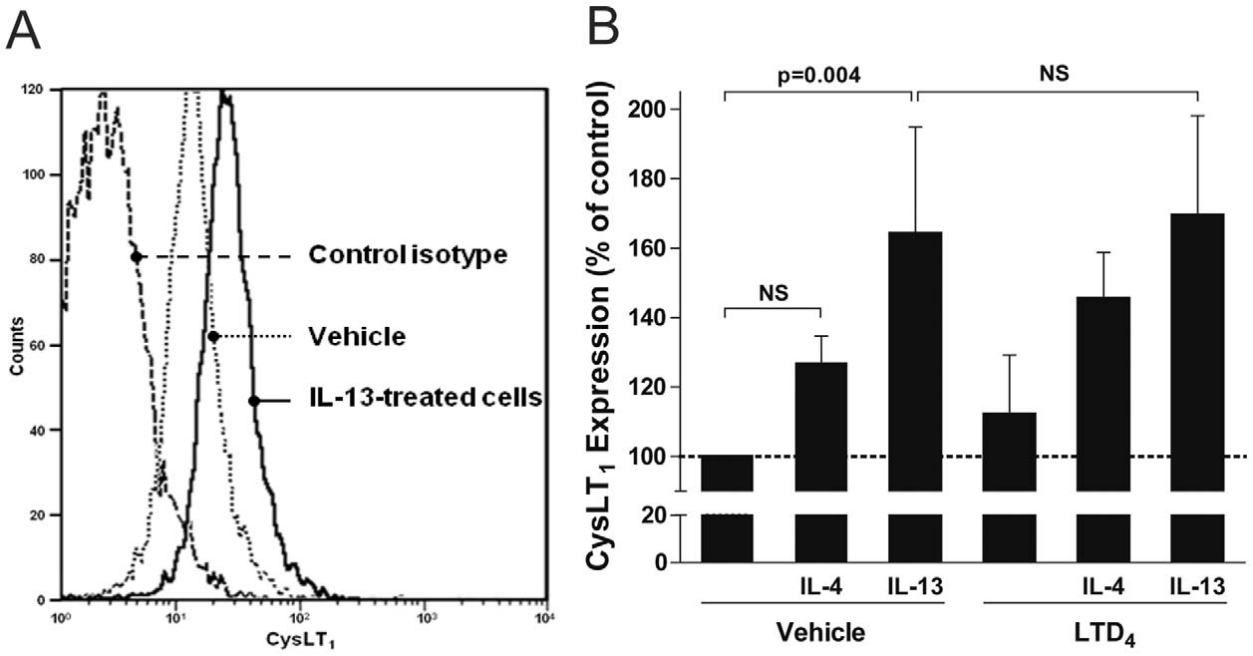
Effect of IL-13, IL-4 and/or LTD_4_ on CysLT_1_ expression by airway epithelial cells. Airway epithelial cells were treated with vehicle, 10 ng/ml IL-4, 10 ng/ml IL-13 and/or 100 nM LTD_4_ for 6 hours. Incubations were stopped by removing the incubation media and cells were harvested, processed, and analyzed for CysLT_1_ expression by flow cytometry as indicated in *Material and Methods*. A. Representative histograms of CysLT_1_ expression (control isotype, open bar; baseline expression, gray plot; IL-13-treated airway epithelial cells). B. Data represent the mean (± SEM) of three separate experiments. NS = not significant.

The puzzling discrepancy between the effects of LTD_4_ on IL-4- and IL-13-induced eotaxin-3 release by airway epithelial cells prompted us to investigate the putative underlying mechanism that could explain this difference. Since airway epithelial cells only express the mRNA for CysLT_1_ (Figure 4), we investigated whether IL-4 and IL-13 had an impact on CysLT_1_ cell surface expression which could explain, at least in part, the differential effect of LTD_4_ on eotaxin-3 release observed in IL-4 or IL-13-activated airway epithelial cells. Flow cytometry analyses were therefore performed on airway epithelial cells following a 6 hours incubation with IL-4 or IL-13. Figure 5 shows that IL-13 significantly increased CysLT_1_ cell surface expression whereas IL-4 was not significantly different from either vehicle or IL-13. The results obtained in these experiments are in line with the effects of LTD_4_ on eotaxin-3 release presented in Figure 3.

**Figure 6:**
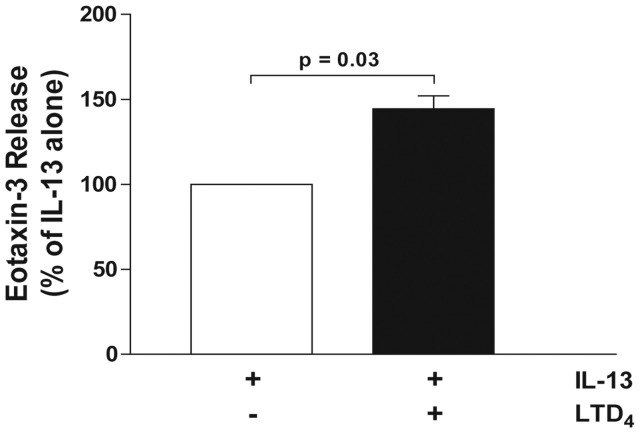
Effect of LTD_4_ on eotaxin-3 release by airway epithelial cells pretreated with IL-13. After a 6 hour incubation of airway epithelial cells with 10 ng/ml IL-13, cells were further cultured for an additional 18 hours in the presence or absence of 100 nM LTD_4_. Incubations were stopped by removing the supernatants and eotaxin-3 was quantified as described in *Material and Methods*. The data represent the mean (± SEM) of three independent experiments.

The data presented herein indicate that the upregulation of CysLT_1_ expression by IL-13 at early timepoints (6 hours) might play a role in the potentiating effect of LTD_4_ on eotaxin-3 release by activated epithelia cells, which occurs at later time points (24 hours). To confirm this hypothesis and since the half-life of LTD_4_ was very short in our experimental model (Figure 3B), we performed experiments in which LTD_4_ was added following a 6 hour treatment of airway epithelial cells with IL-13, i.e. when CysLT_1_ expression is increased. As shown in Figure 6, LTD_4_ also increased the release of eotaxin-3 by airway epithelial cells treated with IL-13 for 6 hours. The effect of LTD_4_ was similar to what we observed when LTD_4_ was added simultaneously with IL-13 (Figure 3).

**Figure 7:**
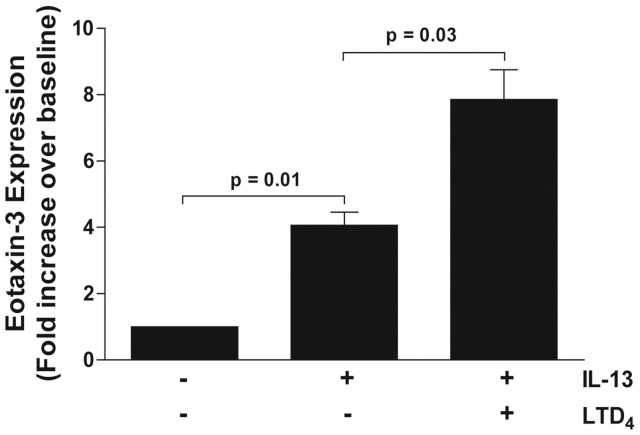
Expression of eotaxin-3 by human primary bronchial epithelial cells. Human primary epithelial cells were treated with vehicle, IL-13 (10 ng/ml) alone or in combination with LTD_4_ (100 nM) for 6 hours. Total RNA was extracted and the expression of eotaxin-3 mRNA was then analyzed by qPCR as described in *Material and Methods*. Eotaxin-3 expression was corrected for level of 18s rRNA as housekeeping gene. Data are presented as fold change over vehicle treatment. Data represent the mean (± SEM) of cell lines from four different subjects in duplicates.

We performed additional experiments in which human primary bronchial epithelial cells were incubated with IL-13 alone or in combination with LTD_4_ for 6 hours, and then eotaxin-3 expression were analysed by qPCR. As shown in Figure 7, the incubation of human primary bronchial epithelial cells with IL-13 for 6 hours induced a 3-fold increase of eotaxin-3 expression. The combination of IL-13 and LTD_4_ amplified the expression of eotaxin-3 induced by IL-13 by 10 times. This potentiating effect of LTD_4_ on eotaxin-3 gene expression from human primary bronchial epithelial cells is consistent and more pronounced than the LTD_4_ effect on IL-13-induced eotaxin-3 release from A549 airway epithelial cells (Figure 3). This supports a potentiating effect of LTD_4_ on the expression of eotaxin-3 induced by IL-13 in human primary bronchial epithelial cells and suggests that both IL-13 and LTD_4_ participate in the accumulation of eotaxin-3 in the lungs, which might be relevant in the physiopathology of asthma.

## Discussion

This study shows that both IL-4 and IL-13 stimulate the release of eotaxin-3 by airway epithelial cells, which is in complete agreement with earlier studies [2], [32], [33]. We also present novel findings showing that LTD_4_ works in concert with IL-13, resulting in an amplification of expression and release of eotaxin-3 by airway epithelial cells most likely through the upregulation of CysLT_1_ expression by IL-13.

Eotaxin-1 is principally produced by leukocytes and to a lesser extent by epithelial or endothelial cells. In contrast, eotaxin-2 and eotaxin-3 are mainly, if not almost exclusively, released by epithelial and endothelial cells and in much greater amount than eotaxin-1 [2], [6], [34]. Moreover, this cell type-specific regulation of eotaxins suggests a site-dependent function of these chemokines. Because of their localization in the bronchial mucosa, activated airway epithelial cells are likely implicated in eosinophilic inflammation by creating a chemotactic gradient of eotaxin-3 that attracts circulating and tissue eosinophils toward the airway lumen.

Moreover, the expression of each eotaxin is differentially regulated. For example, the production of eotaxin-1 is triggered by T_H_1 and T_H_2 cytokines, whereas that of eotaxin-3 is only modulated by T_H_2 cytokines [7], [8]. These differences probably account for their non-redundant roles *in vivo*. Eotaxin-1 could be secreted at inflammatory sites during the early phase of the allergic immune response where both T_H_1 and T_H_2 cytokines are present. In contrast, eotaxin-3 might be preferentially secreted at later time points when a T_H_2-dominant inflammatory state is observed. This hypothesis is supported by studies demonstrating that eotaxin-3, but not eotaxin-1, expression in bronchial mucosa is increased after allergen challenge in asthmatic individuals [35], [36].

CysLTs are not on mighly potent bronchoconstrictors [37] as they are also implicated in the regulation of gene expression in numerous cell types [18]–[28]. This latter role of cysLTs has indeed been observed in epithelial cells where LTD_4_ can upregulate the expression of the *MUC2* gene and transforming growth factor-β1 in a CysLT_1_-dependent manner [38], [39]. Importantly, the regulation of gene expression by cysLTs is often limited and regularly requires a co-stimulus [19]–[28]. For example, while having no effect by themselves, cysLTs increase the production of collagen, eotaxin-1 and CCL2 by transforming growth factor-β1-activated fibroblasts, IL-13-treated fibroblasts, and IL-4-treated monocytes, respectively [40]–[42]. In the present study, we showed that LTD_4_ alone did not induce the release of eotaxin-3 from airway epithelial cells, but significantly augmented the effect of IL-13 but not IL-4 (Figure 3), in agreement with the above-mentioned studies. The transcription of the eotaxin-3 gene induced by IL-4 or IL-13 involves the specific transcription factor signal transducer and activator of transcription (STAT) 6 [7], [43]–[45]. The activity of this transcription factor is increased following its phosphorylation by janus kinases (JAK). STAT6 activation involves JAK1, JAK2, JAK3 and Tyk2, depending on which receptor is activated by IL-4 or IL-13 [31]. The activity of JAK proteins is negatively regulated by the suppressors of cytokine signalling (SOCS) proteins [31]. A recent study even reported that SOCS-1 and SOCS-3 are involved in the transcription of the eotaxin-3 gene [46]. Serezani and colleagues recently demonstrated that LTB_4_ enhanced the expression of MyD88 by downregulating SOCS-1 expression in murine alveolar macrophages [47]. The enhanced mRNA levels (Figure 7) we observed using the combination of LTD_4_ and IL-13 (compared to IL-13 alone) is in line with the above mentioned studies and suggest that LTD_4_ might also enhance eotaxin-3 translation by downregulating SOCS proteins. This however fosters additional studies to determine and confirm whether this occurs in our experimental model.

Since LTD_4_ mainly activate the CysLT_1_ and CysLT_2_ receptors [11], [12], we evaluated their expression by airway epithelial cells. We found that resting airway epithelial and human primary bronchial epithelial cells only expressed CysLT_1_ (Figure 4), indicating that the effect of LTD_4_ on eotaxin-3 release is likely the consequence of CysLT_1_ receptor activation. Even though both IL-4 and IL-13 were previously shown to up regulate the expression of CysLT_1_ [15], [16], [41], [42], [48], the potentiating effect of LTD_4_ on eotaxin-3 accumulation and CysLT_1_ expression was only significant could only be observed in IL-13-stimulated cells (Figure 3). Interestingly, while IL-13 upregulates CysLT_1_ expression on both leukocytes and structural cells (e.g. fibroblasts, smooth muscle and epithelial cells), IL-4 has only been described to do so in leukocytes [15], [16], [41], [42], [48]. Given that airway epithelial cells are structural cells, this might reflect the expression of distinct IL-4 and IL-13 receptor chains in airway epithelial cells. In this respect, the IL-4Rα and IL13Rα1 subunits were shown to be expressed cells by A549 cells [49] and human primary bronchial epithelial cells [50], [51], whereas the common γ chain and the IL-13Rα2 are expressed by human primary bronchial epithelial cells [51], [52]. Importantly, these different receptor subunits differentially interact with JAK proteins (the target of SOCS) [53]. The putative regulatory role LTD_4_ might play at regulating SOCS proteins described above could explain why LTD_4_ potentiated eotaxin-3 expression upon treatment with IL-13 but not IL-4. This again foster additional studies to elucidate whether this regulatory mechanism is also involved for the differential regulation of CysLT_1_ receptor expression between structural cells and leukocytes.

The synergistic effect between LTD_4_ and IL-13 on eotaxin-3 release was only observed after 24 hours of incubation when eotaxin-3 levels remained moderate but not after 48 hours when eotaxin-3 levels are very high. A loss of function of either IL-4 or IL-13 is unlikely since the accumulation of eotaxin-3 induced by these cytokines requires a continuous exposure to the cytokines and can be observed for up to 96 hours [2]. We did not perform experiments beyond 48 hours given our cells cultures were forming multiple layers after 48 hours. The ineffectiveness of LTD_4_ at later time points is likely explained by the short half-life of LTD_4_ we observed, suggesting that LTD_4_ acts rapidly upon its addition in the incubation media, its effect fading away at later time points.

In conclusion, the results presented in this study demonstrate a cooperation between LTD_4_ and IL-13 in the expression and the release of eotaxin-3 by airway epithelial cells. Both mediators could be seen as exerting a positive effect on the other, leading to a cascade of events amplifying the release of eotaxin-3. First, IL-13 rapidly activates numerous transcription factors involved in eotaxin-3 and CysLT_1_ receptor expression (Figure 5 and 7) while LTD_4_ activates CysLT_1_ on airway epithelial cells, resulting in an amplification of eotaxin-3 mRNA transcription and protein release possibly by SOCS inhibition (Figure 6 and 7).

Given that eotaxin-3 levels and CysLT_1_ expression are elevated in bronchial epithelial cells from asthmatics [1], [17] and that the biosynthesis of cysLTs is increased in asthma, we propose a new mechanism that could be implicated in the physiopathology of this disease. First, elevated levels of T_H_2 cytokines such as IL-13 in the bronchial mucosa increase the expression of CysLT_1_ by epithelial cells and, consequently, their responsiveness to cysLTs. When cysLTs are released by activated tissue leucocytes (mast cells and eosinophils), this activate CysLT_1_ on airway epithelial cells and, consequently, augment the release of eotaxin-3 induced by IL-13 which then activates pro-inflammatory functions of eosinophils, including the biosynthesis of cysLTs [54]. Further *in vivo* studies should be performed to confirm if this cycle would amplify and worsen the inflammatory state observed in asthma.

## Materials and Methods

### Reagents and cells

Human recombinant IL-4 and IL-13 were obtained from Peprotech Inc., Rocky Hill, NJ, USA; LTD_4_ and rabbit polyclonal anti-human CysLT_1_ receptor from Cayman Chemical, Ann Arbor, MI, USA; rabbit Immunoglobulin Negative Control from Dako Canada Inc., Mississauga, ON, Canada; eotaxin-1, eotaxin-2 and eotaxin-3 Quantikine Kits from R&D Systems Inc., Minneapolis, MN USA; RNeasy Plus Mini Kit, One Step RT-PCR Kit, RT^2^ First Strand Kit and RT^2^ SYBR Green ROX qPCR Mastermix from Qiagen Inc., Mississauga, ON, Canada; Alexa Fluor® 488 goat anti-rabbit IgG antibody from Invitrogen Canada Inc., Burlington, ON, Canada; bovine serum albumin (BSA), foetal bovine serum (FBS), Hank's buffered salt solution (HBSS) and Dulbecco's Modified Eagle's Medium (DMEM) from WISENT Inc., St-Bruno, QC, Canada. Human primary bronchial epithelial cells isolated from bronchial biopsies obtained from four subjects [55]. Human blood eosinophils were purified from three subjects by negative selection as previously described [56]. All subjects signed an informed consent form for the study with the approval from the local ethic committee named *Comité d'éthique de la recherche de l'Institut universitaire de cardiologie et de pneumologie de Québec*. A549 airway epithelial cells were purchased from the ATCC (Manassas, VA, USA).

### Airway epithelial cell culture, stimulation and analysis of eotaxins

A549 airway epithelial cells were cultured in DMEM supplemented with 10% FBS. Cells were grown in 6-well plates (10^5^ cells/well) until they reached 80% confluence, then serum-deprived overnight before stimulation. Airway epithelial cells were next incubated with IL-4 (10 ng/ml) or IL-13 (10 ng/ml) alone or in combination with LTD_4_ (100 nM) for 6, 24 and/or 48 hours. In selected experiments (Figure 6), airway epithelial cells were incubated with IL-13 alone for 6 hours, followed by the addition of 100 nM LTD_4_ for an additional 18 hours. Incubations were stopped by harvesting the supernatants. Supernatants were immediately spun to remove cell debris, aliquoted and kept at −80°C until the analysis of eotaxins by ELISA according to the manufacturer's instructions. Following the removal of supernatants, cells were immediately harvested and processed for either RT-PCR or flow cytometry analyses.

### Analysis of cysLT receptors by RT-PCR

Total RNA was extracted from purified human blood eosinophils, primary human bronchial epithelial cells and A549 airway epithelial cells using the RNeasy Plus Mini Kit. Both cDNA synthesis and PCR amplification were performed in a single tube using the One Step RT-PCR Kit. RT-PCR reactions were carried out on a Peltier Thermal Cycler PTC-200 (MJ Research, Watertown, USA). Specific primer for CysLT_1_ (NM_00663, forward; 5′-GCCATGACACTATTGATGACTTCCGC-3′, reverse; 5′-CGGTCACGACCATGATCATTCCTATAGC-3′, 678 pb) and CysLT_2_ (NM_020377.2, forward; 5′-CCTTCAGCAATAACAACAGC-3′, reverse; 5′-CCAGATTTAGCATGAAAACG-3′, 185 pb) were used. RT-PCR settings were: denaturation (94°C, 30 sec (15 min for the first cycle), annealing (65°C, 30 sec), and extension (72°C, 1 min (10 min for the last cycle). After amplification, the PCR products were analyzed by electrophoresis on agarose gels as described previously [56].

### Analysis of CysLT_1_ expression by flow cytometry

The expression of CysLT_1_ by airway epithelial cells was assessed by flow cytometry as described before [15]. In brief, cells were washed with PBS, then fixed and permeabilized with the Cytofix/Cytoperm™ solution (BD Biosciences, Mississauga, ON Canada) for 20 min at 4°C. Cells were resuspended in cold (4°C) HBSS containing 1% BSA and 0.1% saponin and incubated at 4°C for 45 min with the rabbit anti-human CysLT_1_ antibody or the appropriate control isotype. Cells were then washed with cold (4°C) HBSS containing 1% BSA and incubated at 4°C for 30 min with the Alexa Fluor® 488 goat anti-rabbit IgG antibody. Cells were washed again and finally resuspended in HBSS. Flow cytometric analysis was performed with an EPICS XL-MCL flow cytometer (Beckman-Coulter, Miami, FL).

### Analysis of LTD_4_ and LTE_4_ by reversed-phase HPLC

A549 cells at 80% confluence were incubated with 100 ng of LTD_4_ in phenol red-free DMEM supplemented with 10% FBS for different times (see figure 3 legend). Incubation were stopped by adding 1 volume of cold (4°C) incubation medium. Samples (cells and media) then were harvested and denatured overnight with 0.5 volume of a cold (−20°C) stop solution (methanol/acetonitrile, 1/1) containing 12.5 ng of both 19-OH-PGB_2_ and PGB_2_ as internal standards. Samples then were centrifuged and the supernatants were collected for the analysis of cysLTs by reversed-phase HPLC as described before [57].

### Analysis of eotaxin-3 expression by qPCR

Human primary bronchial epithelial cells were obtained as previously described [55]. Cells were cultured in DMEM/F12 supplemented with 10% FBS in 12-well plates until they reached 80% confluence. Human primary bronchial epithelial cells were next incubated with IL-13 (10 ng/ml) alone or in combination with LTD_4_ (100 nM) for 6 hours in DMEM/F12 with 1% FBS. Following its extraction with the RNeasy Mini Kit, total RNA (200 ng) will be converted into cDNA with Peltier Thermal Cycler PTC-200 using the RT^2^ First Strand Kit including built-in positive control elements for the detection of genomic DNA contamination and the efficiency of the reverse transcriptase and the polymerase chain reactions. qPCR amplification were done with commercially eotaxin-3 primers (Human CCL26 QuantiTect Primer Assays, NM_006072, from Qiagen Inc.) and RT^2^ SYBR Green ROX qPCR Mastermix on 7900HT Fast Real Time PCR System (Applied Biosystems Canada, Streetsville, ON). A typical PCR amplification will be as follows: a denaturation step at 95°C for 5 min followed by 40 cycles (1-min denaturation at 95°C, 30-sec annealing at 60°C, and 1-min elongation at 72°C). Data acquisition and analyses will be performed with the SDS software (version 2.3). Eotaxin-3 expression was corrected for levels of 18 s rRNA as housekeeping gene.

### Statistical analyses

Means and SEM were determined for continuous variables. The effect of IL-13 on the release of eotaxins from airway epithelial cells was analyzed using Dunnett's method. The data obtained from kinetic experiments and qPCR analyses were evaluated by a Tukey-Kramer's method. The cooperation of LTD_4_ with IL-13 on the release of eotaxin-3 and the expression of the CysLT_1_ receptor by flow cytometry were compared by using 2-way randomized block design. The results were considered significant if *P* values were <0.05. The data were analyzed using the statistical package program SAS version 9.1.3 (SAS Institute Inc., Cary, NC).
